# Protective Effect of L-Carnitine and Coenzyme Q10 on CCl_4_-Induced Liver Injury in Rats

**DOI:** 10.3797/scipharm.1006-02

**Published:** 2010-08-23

**Authors:** Sanaa Ahmed Ali, Lilla Faddah, Ateff Abdel-Baky, Asmaa Bayoumi

**Affiliations:** 1 National Research Centre, El-Behooth St. 12622 Dokki, Giza, Cairo, Egypt; 2 King Saud University, KSA, 11495 Ryad, Zip code 22480, Saudi Arabia; 3 El-Minia University, Faculty of Pharmacy, Salah Salem St. 61519, Egypt

**Keywords:** L-carnitine, Coenzyme Q_10_, Carbon tetrachloride, Liver, Serum, Rats

## Abstract

This study provides an information about the mechanisms of liver injury induced by CCl_4_, and determines the influence of administration of L-carnitine or/and CoQ10 as prophylactic agents against CCl_4_ deteriorative effect. The study was carried out on 80 adult male albino rats divided into eight groups, 10 animals each, as follows: four normal groups (control, treated with L-carnitine, treated with CoQ10, and treated with a combination of Lcarnitine and CoQ10) and four liver injury groups treated with CCl_4_ (control, treated with L-carnitine, treated with CoQ10, and treated with a combination of L-carnitine and CoQ10). Liver injury was induced by s.c. injection of a single dose of CCl_4_ (1 ml/kg). L-carnitine (50 mg/kg/day) was given i.p. for four successive days 24 hours before CCl_4_ injection, and CoQ10 (200 mg/kg) was given as a single i.p. dose 24 hours before CCl_4_ injection. Animals were sacrificed 24 hours after CCl_4_ injection, blood samples were withdrawn and liver tissue samples were homogenized. The levels of the following parameters were determined: hepatic reduced glutathione, serum ALT and AST, hepatic lipid peroxides, hepatic vitamin C, hepatic and serum total protein, serum albumin, serum sialic acid, serum nitrite, and serum and hepatic total LDH activities and LDH isoenzymes. The obtained data revealed that CCl_4_ injection produced a significant decrease in reduced glutathione content, vitamin C, total protein and albumin levels. However, there was a significant increase in serum ALT and AST activities, lipid peroxides, sialic acid, nitric oxide, serum and hepatic total LDH activities. On the other hand, groups treated with L-carnitine or/and CoQ10 prior to CCl_4_ injection showed an improvement in most parameters when compared with cirrhotic control group. It has been concluded that L-carnitine and coenzyme Q10 have a pronounced prophylactic effect against liver damage induced by halogenated alkanes such as carbon tetrachloride.

## Introduction

The use of many halogenated alkanes such as carbon tetrachloride (CCl_4_), chloroform (CHCl_3_), or iodoform (CHI_3_) has been banned or severely restricted because of their distinct toxicity. Yet CCl_4_ continues to provide an important service today as a model substance to elucidate the mechanisms of action of hepatotoxic effects such as fatty degeneration, fibrosis, hepatocellular death and carcinogenicity [1, 2].

**Fig. 1.**
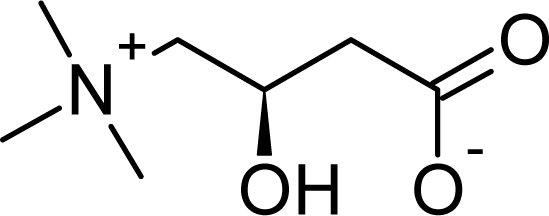
Structure of L-Carnitine

L-carnitine is 4-trimethylamino-3-hydroxybutyrate (Fig. 1). It is an amino acid derivative that is found in nearly all cells of the body and occurs naturally in animal products. It is synthesized chiefly in the liver and kidneys from the essential amino acid L-lysine [3]. Liver is one of the main sources of endogenous carnitine synthesis from lysine, methionine, ascorbate, niacin, pyridoxine and Fe^2+^. Carnitine is captured and stored by muscle because there is no carnitine synthesis in muscle [4]. Carnitine deficiencies may cause symptoms such as fatigue, chest pain, muscle pain, weakness, low blood pressure, and/or confusion. A healthcare provider may recommend use of the supplement levocarnitine (L-carnitine) for individuals who have a suspected or confirmed deficiency of this nutrient.

**Fig. 2.**
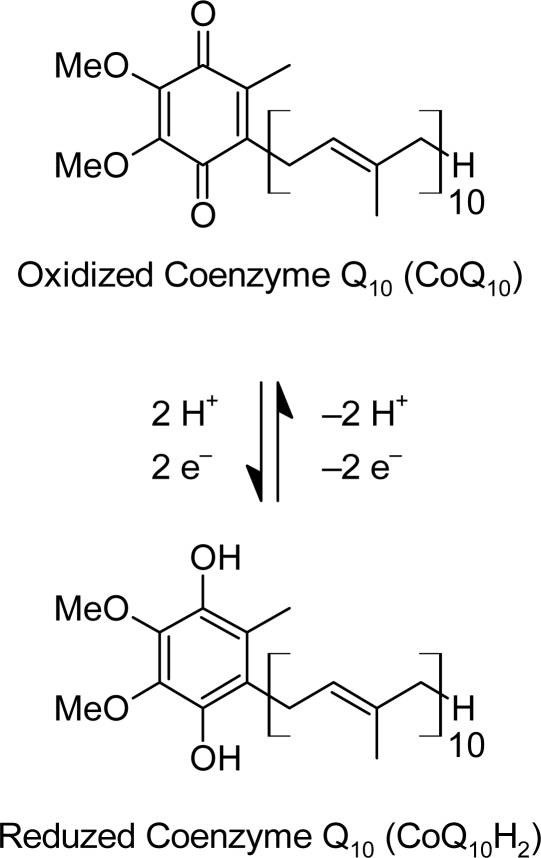
The Redox Transitions of CoQ_10_

Coenzyme Q_10_ (CoQ_10_) or ubiquinone is a vitamin-like substance which is found in small amounts in a wide variety of foods and is synthesized in all tissues. CoQ_10_ is the coenzyme for at least three mitochondrial enzymes (complexes I, II and III). Mitochondrial enzymes of the oxidative phosphorylation pathway are essential for the production of the high-energy phosphate containing compound, adenosine triphosphate (ATP), upon which all cellular functions depend [5]. Coenzyme Q besides its bioenergetic function in mitochondrial respiratory chain is a powerful lipid-soluble antioxidant synthesized in the liver [6]. Protective effects of vitamin E and coenzyme Q administration found in experimental models of CCl_4_-induced cell necrosis support the role of free radicals in liver damage [7]. The structures of CoQ_10_ and its redox transitions are shown in Fig. 2.

The aim of this work is to evaluate the prophylactic effect of L-carnitine, coenzyme Q_10_ (CoQ_10_) or their combination against the deteriorative effect induced by carbon tetrachloride (CCl_4_) on serum & liver. Administration of CCl_4_ provides a suitable animal model for free radical damage in liver such as fatty degeneration, fibrosis and hepatocellular death.

## Results

CCl_4_ injection produced a significant decrease in hepatic reduced glutathione (GSH); Ascorbic Acid (Vit C); total protein content as compared to normal control value. Administration of L-carnitine or/and coenzyme Q_10_, significantly increased these parameters compared to CCl_4_ control group as shown in Tables 1, 2. The percentages of improvement were 28.79%, 27.11% and 29.51%;(GSH) 16.42%, 27.55% and 29.91% (Vit C); 16.3%, 22.59% and 28.6% (total protein) on injection of L-carnitine, coenzyme Q_10_ and their combination respectively. Thus, combination has achieved the most liver protection. Also, CCl_4_ cause a significant decrease in serum total protein level; serum albumin compared to normal control value. Administration of L-carnitine or/and coenzyme Q_10_ (prior to CCl_4_ injection) significantly increased serum total protein and albumin levels as compared to CCl_4_ control group as shown in Table 1. The percentages of improvement were 44.43%, 65.29% and 67.53% (serum total protein) 14.17%, 10.9% and 21.65% (albumin) on injection of L-carnitine, coenzyme Q_10_ and their combination respectively. Thus, combination has achieved the most liver protection.

While, showed a significant increase in serum ALT& AST activities as compared to normal control value. After treatment, significantly decreased in the two enzymes activities compared to CCl_4_ control group as shown in Table 1 the percentages of improvement were 26.94%, 56.12% and 56.7 % (ALT) 26.69%, 31.13% and 36.15 % (AST) on injection of L-carnitine, coenzyme Q_10_ and their combination respectively. Thus, combination has achieved the most liver protection. Also, CCl_4_ showed a significant increase in serum sialic acid; nitrite levels; total LDH activity and hepatic malondialdehyde (LPO) level compared to normal control value. Administration of any one of the three compounds prior to CCl_4_ injection) significantly decreased in all mentioned parameters compared to CCl_4_ control group as shown in Table 2.

The percentages of improvement were 66.18%, 77.57% and 78.81% sialic acid, 26.6%, 16.98% and 52.08% (nitrite level) 55.02%, 72.34% and 99.85% total LDH activity and 86.25%, 97.55% and 94.63% hepatic malondialdehyde level injection of L-carnitine, coenzyme Q_10_ and their combination respectively. Thus, Coenzyme Q10 is more effective than L-carnitine in most parameters as AST, Sialic Acid, serum & hepatic total protein, serum &hepatic LDH,lipid peroxide and vitamin C and the best effects of the combination has achieved the most liver protection as shown in tables 1 and 2.

### Separation of LDH Isoenzymes by Gel Electrophoresis Hepatic Total Lactate Dehydrogenase (LDH) Activity

CCl_4_ injection produced a significant increase in hepatic total LDH activity at P < 0.05 compared to normal control value. Administration of L-carnitine or/and coenzyme Q_10_ (prior to CCl_4_ injection) significantly decreased hepatic total LDH activity compared to CCl_4_ control group as shown in Table 3. The percentages of improvement were 15.79%, 23.33% and 41.23% on injection of L-carnitine, coenzyme Q_10_ and their combination respectively. Thus, combination has achieved the most liver protection.

**Fig. 3.**
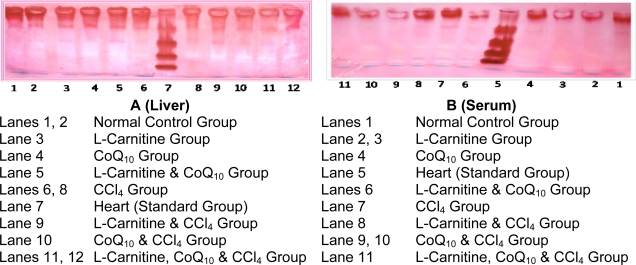
Electrophoretic Profile of Lactate Dehydrogenase Isoenzymes (LDH-Isoenzymes) of Male Rats (Liver & Serum).

Fig. 3A. shows the electrophoretic separation pattern of hepatic lactate dehydrogenase isoenzymes in normal and CCL_4_ groups. It can be observed that hepatic LDH-1 was absent in all groups. CCl_4_ injection resulted in a significant increase in hepatic levels of LDH-2, LDH-3 and LDH-5; however, it significantly decreased hepatic LDH-4. The administration of any one of the three compounds amelioration all the levels of hepatic LDH Isoenzymes, compared to CCl_4_ group.

Table 4 & Fig. 3B show the electrophoretic separation pattern of serum lactate dehydrogenase isoenzymes in normal and cirrhotic groups. It can be observed that CCl_4_ injection resulted in a significant increase in serum levels of all LDH isoenzymes. Administration of L-carnitine alone significantly decreased serum LDH-1 and LDH-5; however, it increased serum LDH-2, LDH-3 and LDH-4 compared to CCl_4_ control group. Administration of coenzyme Q_10_ alone significantly decreased serum levels of all LDH isoenzymes compared to CCl_4_ control group. On the other hand, administration of a combination of L-carnitine and coenzyme Q_10_ significantly decreased serum LDH-1, LDH-2, LDH-3 and LDH-5; however, it increased serum LDH-4 compared to CCl_4_ control group.

## Discussion

Carbon tetrachloride is widely used as an animal model of liver damage which is caused by formation of trichloromethyl and trichloromethylperoxyl radicals, initiating lipid peroxidation and resulting in fibrosis and cell necrosis [8]. Kodai et al. [9] reported that there is an increased serum level of MDA in CCl_4_-induced acute hepatic damage. The formation of malondialdehyde was significantly decreased in antioxidant-treated groups compared to CCl_4_-treated group. CCl_4_ administration induced lipid peroxidation accompanied by increases in the plasma malondialdehyde [2, 10]. In this study, CCl_4_ injection produced a significant increase in liver LPO level (expressed in terms of malondialdehyde). L-carnitine or/and coenzyme Q_10_ (prior to CCl_4_ injection) significantly decreased hepatic LPO level. Citil et al. [11] suggested that L-carnitine brought about the inhibition of lipid peroxidation by enhancing antioxidant capacity.

Frei et al. [12] showed that ascorbic acid was effective in inhibiting lipid peroxidation by efficiently trapping peroxyl radicals before they can initiate lipid peroxidation. It can also protect biological membranes against peroxidative damage, either directly or via enhancing the activity of α-tocopherol by maintaining it in its reduced active form. Niki et al. [13] have observed a decreased plasma level of vitamin C in cirrhosis stage. CCl_4_ injection produced a significant decrease in hepatic vit C content. Administration of the two compounds (prior to CCl_4_ injection) significantly increased hepatic vit C content, the most improvement has been achieved by the combination.

A significant decrease in the level of hepatic reduced glutathione content in CCl_4_ intoxicated rat, these results are in line with the previous studies of Wills and Asha [10] have reported a significant reduction in hepatic glutathione content in CCl_4_-induced hepatic damage in rats. Decreased concentration of GSH level could also be a result of depression of ATP synthesis and acidosis due to anaerobic glycolysis, Over 90% of GSH inflow in systemic circulation is accounted for by the influx of this peptide from the liver [14]. This study is in accordance with the above mentioned results and shows that CCl_4_ injection produced a significant decrease in hepatic glutathione content, L-carnitine and coenzyme Q_10_ significantly increased hepatic glutathione content

Our study, liver function tests performed for CCl_4_ intoxicated rats showed an increase in the activities of ALT and AST enzymes. These results are supported by Allis et al. [15]. It is observed that administration of L-carnitine or/and coenzyme Q_10_ significantly decreased serum ALT and AST activities. Also, Sumimoto et al. [16] have reported that ischemically damaged livers pretreated with CoQ_10_ showed a decrease in the activity of serum ALT and AST.

CCl_4_ injection produced a significant decrease in hepatic total protein, this result was previously reported by Allis et al. [15]. L-carnitine and coenzyme Q_10_ treatment significantly increased hepatic total protein compared to CCl_4_ treated group. In accordance with these findings, Sumimoto et al. [16] found that ischemically damaged liver that was pretreated with CoQ_10_ showed an increase in levels of total protein to the normal range. As most blood proteins are synthesized in the liver, decreased levels can be found in liver injury because of decreased liver synthesis. Naik and Panda [17] have observed a decreased serum level of total protein in CCl_4_ induced hepatotoxicity in rats. This level was elevated by giving antioxidants prior to CCl_4_ administration. Also, Wang et al. [18] have reported that there is a decreased serum level of total protein in CCl_4_-induced acute hepatic damage. The treatments with antioxidants elevated total protein levels significantly. In this work, CCl_4_ injection produced a significant decrease in serum total protein level Administration of either the two compounds significantly increased serum total protein level compared to CCl_4_ group. The liver produces all of the proteins except for the proteins synthesized by the immune system (called gamma globulin or immunoglobulin), it does this by reassembling amino acids into protein. The main protein produced by the liver is albumin [19].

Normal albumin in the bloodstream is important for many physiologic functions; one of these functions involves the normal maintenance of fluid pressure in the arteries and veins. When the protein level falls below a certain point, the fluid in these vessels can leak out and pool in the abdominal or thoracic cavities “ascites”. Determining serum albumin level is often considered as a test for liver function. This is mainly because hepatic synthesis of albumin tends to decrease in end-stage liver disease [20]. In this study, CCl_4_ caused significant decrease in serum albumin level, Administration of the two compounds or their mixture significantly increased serum albumin level.

Variation in serum sialic acid level in a variety of inflammatory liver diseases is an important diagnostic and prognostic tool [21]. In liver destruction, its level rises proportionally to hepatic damage because much of the circulating sialic acid is covalently attached to glycoproteins and more than 50% of total sialic acid comes from acute phase proteins such as alpha acid glycoproteins, alpha anti-trypsin and fibrinogen, factor VII antigen and activation markers of coagulation [22].

Several mediators of systemic vasodilatation in liver cirrhosis have been reported. Among these is nitric oxide (NO), which has been proposed as one of the main mediators. Tiptoe et al. [23] proposed that a high level of nitric oxide is associated with CCl_4_-induced acute liver injury, the study of Nasser et al. [24] showed that serum nitrite levels in cirrhotic patients were significantly (P < 0.05) increased in comparison with the controls. In this study, the administration of coenzyme Q_10_ alone did not lead to a significant decrease in serum nitrite level compared to CCl_4_ control group. The most improvement has been achieved by the combination.

Lactate dehydrogenase, in anaerobic glycolysis, catalyzes the conversion of pyruvate to lactate which subsequently is converted to glucose in gluconeogenic flux. LDH system reflects the NAD^+^/NADH ratio indicated by the lactate/pyruvate ratio of hepatocyte cytosol [25]. The results obtained in this study showed that CCl_4_ injection produced a significant increase in serum total LDH activity. These elevations are reduced by L-carnitine or/and coenzyme Q_10_.

Our data are in harmony with Wills and Asha [10] who reported that CCl_4_ administration in rats caused elevation of serum LDH activity. Koudelova et al. [26] have observed that pretreatment of intoxicated rats with L-carnitine decreased serum LDH activity probably due to membrane stabilizing role of L-carnitine which leads to reduction of LDH leakage from liver. Takeuchi et al. [27] also found that L-carnitine administration significantly reduces LDH leakage from intoxicated primary cultured rat hepatocytes. Takayama et al. [28] have observed that coenzyme Q_10_ lowers the increased level of serum LDH in rats subjected to hepatic ischemia-reperfusion

The electrophoretic separation pattern of serum lactate dehydrogenase isoenzymes in normal and injury groups showed that CCl_4_ injection resulted in a significant increase in all serum levels of LDH isoenzymes. It was noticed that CCl_4_ group showed an abnormal serum LDH isoenzyme pattern, with a significant increase in all isoenzymes. L-carnitine (prior to CCl_4_ injection) resulted in a significant increase in isoenzyme 2, isoenzyme 3 and isoenzyme 4; and a significant decrease in isoenzyme 1 and isoenzyme 5. Treatment with coenzyme Q_10_ (prior to CCl_4_ injection) resulted in a significant decrease in all isoenzymes. Treatment with a combination of L-carnitine and coenzyme Q_10_ (prior to CCl_4_ injection) resulted in a significant increase in isoenzyme 4 and a significant decrease in isoenzyme 1, isoenzyme 2, isoenzyme 3 and isoenzyme 5. Henry and Ferguson [29] reported that LDH-1 isoenzyme is maximally active at low concentration of pyruvate and inhibited by excess pyruvate, while LDH-5 isoenzyme maintains its activity at high pyruvate concentration. In cases of acute viral hepatitis, hepatic LDH concentration is altered in about 50% of the patients, with values typically only slightly above the reference limit [30].

In the present investigation, CCl_4_ injection produced a significant increase in hepatic LDH activity. Administration of L-carnitine or/and coenzyme Q_10_ significantly decreased hepatic total LDH activity compared to CCl_4_ group. The most improvement has been achieved by the combination. Mitcheva et al. [31] reported that CCl_4_ injection leads to a significant increase in LDH leakage from rat hepatocytes. This may be attributed to CCl_4_-induced dehalogenation in the liver endoplasmic reticulum. This process leads to trichlormethyl radical (CCl_3_•) formation and initiation of lipid peroxidation.

The electrophoretic separation pattern of hepatic lactate dehydrogenase isoenzymes in normal and injury groups showed a disturbance in level of hepatic LDH isoenzymes after CCl_4_ injection. It was noticed that CCl_4_ group showed an abnormal hepatic LDH isoenzyme pattern, with absence of LDH-1, increase in LDH-2, LDH-3, LDH-5 and decrease in LDH-4 level. Treatment with L-carnitine (prior to CCl_4_ injection) resulted in a significant increase in isoenzyme 2, isoenzyme 3 and isoenzyme 4, a significant decrease in isoenzyme 5, whereas isoenzyme 1 was still absent. Treatment with coenzyme Q_10_ (prior to CCl_4_ injection) resulted in a significant increase in isoenzyme 2, isoenzyme 3 and isoenzyme 4, a significant decrease in isoenzyme 5, whereas isoenzyme 1 was still absent. Treatment with a combination of L-carnitine and coenzyme Q_10_ (prior to CCl_4_ injection) resulted in a significant increase in isoenzyme 2, isoenzyme 3 and isoenzyme 4, a significant decrease in isoenzyme 5, whereas isoenzyme 1 was still absent.

## Materials and Methods

### Animals

This study was performed on 80 adult male albino rats. The rats were provided by the animal house unit of El-Nasr Pharmaceutical Company, Cairo, Egypt. Their weight ranged from 150–200 gm (∼ 4 months) at the time of experimentation. The animals were housed in an animal house unit, in the Biochemistry Department, Faculty of Medicine, El-Minia University, in individual metabolic cages and allowed free access to standard diet and water. The standard diet was supplied by El-Nasr Pharmaceutical Company. It is composed of 72.2% carbohydrates, 3.4% fats, 19.8% proteins, 3.6% cellulose, 0.5% vitamins and minerals and 0.5% salts. The rats were left for two weeks for acclimatization. All rats were allowed to fast overnight the day before injection.

### Chemicals

The chemical and solvents used were analar quality, product of Merck, Sigma and El-Naser Pharmaceutical Chemical Company, Egypt.

### Study Design

The 80 rats were divided into eight groups, 10 animals each, as follows:

Group I: Normal control group.
Group II: Normal group treated with L-carnitine.
Group III: Normal group treated with CoQ_10_.
Group IV: Normal group treated with L-carnitine and CoQ_10_.
Group V: CCl_4_ group.
Group VI: CCl_4_ group treated with L-carnitine.
Group VII: CCl_4_ group treated with CoQ_10_.
Group VIII: CCl_4_ group treated with L-carnitine and CoQ_10_.

Liver fibrosis was induced by subcutaneous (s.c.) injection of a single dose of CCl_4_ (1 ml/kg) [32]. L-carnitine (50 mg/kg/day) was given i.p. for four successive days 24 hours before CCl_4_ injection [33], and CoQ_10_ (200 mg/kg) was given as a single i.p. dose 24 hours before CCl_4_ injection [34]. Animals were sacrificed 24 hours after CCl_4_ injection, blood samples were withdrawn and liver tissue samples were homogenized and stored at −80 ^○^C. Anesthetic procedures complied with the legal ethical guidelines approved by the Ethical Committee of the Federal Legislation and National Institutes of Health Guidelines in USA were approved by the ethical committee of the National Research Centre in Egypt.

Samples were subjected to the following biochemical analyses:

Determination of hepatic reduced glutathione content; serum alanine aminotransferase activity; serum aspartate aminotransferase activity. Hepatic lipid peroxides level; hepatic ascorbic acid content; serum and hepatic total protein levels; serum albumin level; serum sialic acid level; serum nitrite level; serum and hepatic total LDH activities; Separation of serum and hepatic LDH isoenzymes by gel electrophoresis.

### Preparation of Serum

Each rat was anesthetized with diethyl ether, and then the sublingual vein was cut with fine scissors. The blood was collected by allowing it to drip into a clean centrifuge tube and allowed to clot at room temperature. The serum was separated from the clot by centrifugation at 3000 rpm for ten minutes, and then the non-hemolyzed serum was transferred into a clean tube and stored at −80 ^○^C.

### Preparation of Liver Homogenate

The rat liver was removed, blotted with filter paper and weighed. The liver was homogenized under ice after addition of phosphate-buffered saline using Potter-Elvehjem homogenizer with Teflon pestle to prepare liver homogenate 20% in phosphate-buffered saline. Also, liver homogenate 5% in saline solution and liver homogenate 5% in tris-glycine buffer were prepared. The homogenates were stored at −80 ^○^C.

### Determination of Hepatic Reduced Glutathione Content

Hepatic reduced glutathione was estimated according to the method of Moron et al. [35].

### Determination of Serum Alanine Aminotransferase Activity

Serum ALT was determined according to the method of Reitman and Frankel [36].

### Determination of Serum Aspartate Aminotransferase Activity

Serum AST was determined according to the method of Reitman and Frankel [36].

### Determination of Hepatic Lipid Peroxides Level

Hepatic lipid peroxides (expressed as malondialdehyde) were estimated by thiobarbituric acid as described by Ohkawa et al. [37].

### Determination of Hepatic Ascorbic Acid Content

The method adopted by Jagota and Dani [38] was used for estimation of vitamin C.

### Determination of Serum and Hepatic Total Protein Levels

Serum total protein was estimated according to the method of Bradford [39].

### Determination of Serum Albumin Level

Albumin was estimated according to the method of Doumas et al. [40]

### Determination of Serum Sialic Acid Level

Serum sialic acid was determined using Ehrlich’s reagent, according to the method of and Waters et al. [41].

### Determination of Serum Nitrite Level

Nitrite, a stable end product of nitric oxide radical, is mostly used as an indicator for the production of nitric oxide. Serum nitrite was determined by using Griess reagent, according to the method of Moshage et al. [42].

### Determination of Serum and Hepatic Total Lactate Dehydrogenase Activities

LDH enzyme activity was estimated by the method of Babson and Babson [43]

Lactate dehydrogenase catalyzes the oxidation of lactate to pyruvate, with concomitant reduction of NAD^+^.

### Separation of Serum and Hepatic Lactate Dehydrogenase Isoenzymes by Gel Electrophoresis

The method of Dietz and Lubrano [44] was adopted for the preparation of ultra-thin layer of 5.5% Polyacrylamide gel for separation of LDH isoenzymes.

### Drying

The gel was taken out and placed up on a piece of cellophane sheets soaked in destaining solution and dried on a filter paper. The dry gel can be handled like a piece of paper using a glass plate, blinder and clips [45].

Scanning: For the evaluation, the dry gel was scanned at 575 nm with Ultrascan Laser Densitometer.

### Statistical analyses

All statistical analyses, including the calculations of mean ± S.D., and statistically analyzed using one way analysis of variance (ANOVA) accompanied with post-hoc Significance was set at P < 0.05 level.

## Conclusion

From the previous results, it has been concluded that L-carnitine and coenzyme Q_10_ have a pronounced prophylactic effect against hepatotoxicity induced by halogenated alkanes such as carbon tetrachloride. Finally, it can be said that the combination of L-carnitine and CoQ_10_ is recommended for protecting the liver from CCl_4_ hazards.

## Figures and Tables

**Tab. 1.** Levels of ALT, AST, albumin, Nitrate, Sialic acid, total protein and LDH activity in the serum of different groups.

**Parameter**	**Normal Groups**				**CCL**_**4**_**-Injury groups**			

	**Contr. (I)**	**L-Carn. (II)**	**CoQ**_**10**_ **(III)**	**L-Carn. + CoQ**_**10**_ **(IV)**	**Contr. (V)**	**L-Carn. (VI)**	**CoQ**_**10**_ **(VII)**	**L-Carn. + CoQ**_**10**_ **(VIII)**
ALT (U/ml)	29.32 ± 2.57	27.18 ± 3.22	16.397* ± 4.82	26.38 ± 3.91	37.49* ± 4.68	29.59# ± 0.69	21.03# ± 1.87	21.05# ± 1.16
AST (U/ml)	71.19 ± 5.16	62.38* ± 3.07	56.45* ± 3.43	59.85* ± 5.40	109.82* ± 7.86	90.82# ± 7.65	87.66# ± 6.06	84.09# ± 3.37
Albumin (gm/dl)	6.17 ± 0.118	6.11 ± 0.19	6.17 ± 0.1338	5.89 ± 0.21	4.72* ± 0.32	5.59# ± 0.22	5.39# ± 0.37	6.06# ± 0.20
Nitrite (μmol/L)	16.01 ± 1.11	7.40* ± 1.64	13.38 ± 2.635	16.64 ± 1.36	26.79* ± 1.04	22.53# ± 2.69	24.07 ± 2.58	18.45# ± 2.00
Sialic Acid (mg/dl)	54.55 ± 4.59	36.86* ± 7.02	45.69 ± 9.383	44.71 ± 9.19	94.59* ± 9.59	58.49# ± 10.97	52.28# ± 7.50	51.6# ± 6.45
Total Protein (mg/ml)	19.3 ± 0.42	17.32 ± 1.26	20.8 ± 0.91	20.8 ± 0.59	5.5* ± 0.14	14.07# ± 1.33	18.1# ± 0.26	18.53# ± 2.00
LDH (μmol/mg protein/min)	81.42 ± 13.47	77* ± 11.76	49.15* ± 14.8	91.14* ± 11.04	209.6* ± 3.94	164.8# ± 10.69	150.7# ± 1.25	128.3# ± 2.04

Data are expressed as mean ± SD of ten rats in each group. Treated normal groups are compared with normal control group CCL_4_-Injury group is compared with normal control group. Treated CCL4 groups are compared with cirrhotic control group. Difference between groups is analyzed by one-way ANOVA, where:

* …significantly different from group I at P < 0.05;

# …significantly different from group V at P < 0.05.

**Tab. 2.** Levels of GSH, Lipid peroxide, Vit. C, total protein, LDH activity in the liver of different groups.

**Parameter**	**Normal Groups**				**CCL**_**4**_**-Injury groups**			

	**Contr. (I)**	**L-Carn. (II)**	**CoQ**_**10**_ **(III)**	**L-Carn. + CoQ**_**10**_ **(IV)**	**Contr. (V)**	**L-Carn. (VI)**	**CoQ**_**10**_ **(VII)**	**L-Carn. + CoQ**_**10**_ **(VIII)**
GSH (mg/gm tissue)	3.93 ± 0.07	5.33* ± 0.88	6.186* ± .92	5.811* ± 1.00	2.507* ± 0.40	3.638# ± 0.19	3.572# ± 0.85	3.67# ± 0.41
Lipid peroxide (nmol/mg protein)	2.568 ± 0.12	2.251* ± 0.18	2.339 ± 0.32	1.683* ± 0.26	5.985* ± 0.27	3.77# ± 0.12	3.48# ± 0.16	3.555# ± 0.11
Vit C (ascorbic acid) (μg/gm tissue)	101.08 ± 8.06	99.47 ± 6.61	90.81 ± 9.34	93.185 ± 11.28	51.26* ± 6.09	67.86# ± 5.52	79.112# ± 1.29	81.49# ± 8.73
Total Protein (mg/gm tissue)	250.11 ± 23.58	258.28 ± 35.71	256.05 ± 8.83	287.96* ± 35.4	176.48* ± 6.23	217.24# ± 14.96	232.99# ± 20.36	248# ± 19.06
LDH (μmol/mg protein/min)	114 ± 1.06	88.9* ± 0.15	99.57* ± 4.43	97.6* ± 1.05	181.9* ± 1.71	163.9# ± 5.73	155.3# ± 1.46	134.9# ± 5.48

Data are expressed as Mean ± SD of ten rats in each group. Difference between groups is analyzed by one-way ANOVA, where:

* …significantly different from group I at P < 0.05;

# …significantly different from group V at P < 0.05.

**Tab. 3.** Total activity of hepatic LDH Isoenzymes.

**LDH Isoenzymes (μmol/mg protein/min)**	**Normal Groups**				**CCL**_**4**_**-Injury groups**			

	**Contr. (I)**	**L-Carn. (II)**	**CoQ**_**10**_ **(III)**	**L-Carn. + CoQ**_**10**_ **(IV)**	**Contr. (V)**	**L-Carn. (VI)**	**CoQ**_**10**_ **(VII)**	**L-Carn. + CoQ**_**10**_ **(VIII)**
LDH-1	–	–	–	–	–	–	–	–
LDH-2	1.01	1.298*	1.47*	1.11*	1.32*	1.72#	2.05#	1.43#
LDH-3	1.85	1.242*	2.99*	3.89*	2.09*	3.74#	2.85#	2.44#
LDH-4	2.60	2.6	9.84*	9.74*	2.29*	16.88#	15.22#	23.29#
LDH-5	108.54	83.76*	85.27*	82.89*	176.20*	141.57#	135.19#	107.74#

Data are expressed as Values are expressed in Mean ± SD of ten rats in each group. Difference between groups is analyzed By one-way ANOVA, where:

* …significantly different from group I at P < 0.05;

# …significantly different from group V at P < 0.05.

**Tab. 4.** Total activity of serum LDH Isoenzymes.

**LDH Isoenzymes (μmol/mg protein/min)**	**Normal Groups**				**CCL**_**4**_**-Injury groups**			

	**Contr. (I)**	**L-Carn. (II)**	**CoQ**_**10**_ **(III)**	**L-Carn. + CoQ**_**10**_ **(IV)**	**Contr. (V)**	**L-Carn. (VI)**	**CoQ**_**10**_ **(VII)**	**L-Carn. + CoQ**_**10**_ **(VIII)**
LDH-1	1.33	1.74*	0.83*	3.14*	6.55*	3.47#	3.86#	3.05#
LDH-2	1.71	1.62*	1.16*	3.72*	7.61*	8.86#	4.49#	3.98#
LDH-3	3.38	4.43*	1.21*	3.66*	4.89*	8.88#	3.83#	3.39#
LDH-4	4.25	6.64*	3.07*	5.59*	5.48*	9.27#	5.35#	5.10#
LDH-5	70.74	62.58*	42.88*	75.02*	185.06*	134.33#	133.16#	111.88#

Data are expressed as Values are expressed in Mean ± SD of ten rats in each group. Difference between groups is analyzed By one-way ANOVA, where:

* …significantly different from group I at P < 0.05;

# …significantly different from group V at P < 0.05.
